# Transcatheter Left Atrial Appendage Occlusion: A Multi-Center Real Life Experience

**DOI:** 10.3390/jcm11236944

**Published:** 2022-11-25

**Authors:** Ziad Arow, Tzipi Hornik-Lurie, Mustafa Gabarin, Alexander Omelchenko, Rami Barashi, Yoav Arnson, Abid Assali, David Pereg

**Affiliations:** 1 Cardiology Department, Meir Medical Center, Kfar Saba 4428164, Israel; 2 Sackler Faculty of Medicine, Tel-Aviv University, Tel-Aviv 6997801, Israel; 3 Meir Medical Center Research Institute, Kfar Saba 4428164, Israel

**Keywords:** atrial fibrillation, left atrial appendage occlusion, ischemic CVA, hemorrhagic CVA, anticoagulation

## Abstract

(1) Background: left atrial appendage occlusion (LAAO) is considered an effective and relatively safe treatment for the prevention of thromboembolic events in patients with atrial fibrillation and a contra-indication for anticoagulation. We present a large multicenter real-world experience of transcatheter LAAO implementation in patients with atrial fibrillation who cannot be treated with chronic anti-coagulation; (2) Methods: included were atrial fibrillation patients who underwent transcatheter LAAO between 1 January 2016 and 30 June 2021. The study was conducted using the electronic health record database of Clalit Health Services (CHS). The primary outcomes included hemorrhagic and ischemic stroke following LAAO; (3) Results: included were 389 atrial fibrillation patients. During a median follow-up of 2.1 years, 13% patients had ischemic cerebrovascular accident (CVA), and 4.4% patients had hemorrhagic CVA. While the risk of ischemic stroke increased gradually over time, the risk of hemorrhagic CVA was highest during the first 3 months following the procedure. Moreover, previous ischemic stroke was the only significant predictor for both hemorrhagic and ischemic stroke following LAAO; (4) Conclusions: while the annual performance rate of transcatheter LAAO has increased significantly over the past years, post procedural long-term prognosis remains poor with a substantial risk of both thrombotic and bleeding events.

## 1. Introduction

Transcatheter left atrial appendage occlusion (LAAO) has been recommended by international guidelines for the prevention of thromboembolic events in patients with atrial fibrillation and a contra-indication for anticoagulation [1,2,3]. Randomized clinical trials have reported high implantation success rates of the different LAAO devices with an acceptable rate of procedure-related complications [2,4,5]. LAAO was non-inferior to vitamin K antagonists (VKAs) or direct oral anticoagulants (DOACs) for stroke prevention with a significant reduction in major bleeding, including hemorrhagic stroke [4,5,6]. However, the available information is based mainly on randomized controlled trials (RCTs) and therefore may be limited by a selection bias. Since patients enrolled in RCTs may not accurately represent a real-life population, additional real-life data regarding the procedure’s safety and efficacy are warranted. The current study presents a large multicenter nationwide real-world experience including long term efficacy and safety of transcatheter LAAO in patients with atrial fibrillation who cannot be treated with chronic anti-coagulation.

## 2. Materials and Methods

This retrospective cohort study was conducted using the electronic health record database of Clalit Health Services (CHS). CHS is the largest Israeli payer–provider integrated health care system serving >4.5 million members, which covers 54% of the Israeli population. The database includes patient demographics and clinical characteristics, hospital and outpatient clinic diagnoses, medical treatments, medication dispensation information and laboratory test results. Data were extracted from Clalit Health Services (CHS) using the Clalit research data sharing platform powered by MDClone (https://www.mdclone.com, accessed on 5 September 2022). All clinical diagnoses were identified using *International Classification of Diseases*, *Ninth Revision* (ICD-9) codes. for the study endpoints we used ICD9 codes only from hospitalizations that occurred following the LAAO hospitalization. The study population consisted of patients >18 years with non-valvular atrial fibrillation who underwent transcatheter LAAO between 1 January 2016 and 30 June 2021. The procedure is approved by the Israeli Ministry of Health for patients with a history of life-threatening bleeding or bleeding while on anti-coagulation treatment with Bleeding Academic Research Consortium (BARC) 3B or 3C and a CHADS2 score ≥2. Standard post procedural treatment included dual anti-platelet therapy with aspirin and clopidogrel for 3 months followed by single anti-platelet therapy (either aspirin or clopidogrel) for life.

The primary outcomes included hemorrhagic and ischemic stroke. Secondary endpoints included all-cause death, gastrointestinal (GI) bleeding requiring blood transfusions, and myocardial infarction (MI) following the procedure.

The study was approved by the local institutional ethics committee in keeping with the principles of the Declaration of Helsinki. In accordance with Ministry of Health regulations, the institutional ethics committee did not require written informed consent since data were collected anonymously from the computerized medical files, with no active participation of patients.

**Statistical analysis:** Descriptive statistics were obtained for all study variables. All categorical variables were presented as absolute numbers and percentages, and continuous variables were presented as mean (SD) or median (interquartile range [IQR]) values as appropriate. Cox proportional-hazards analyses were used to estimate the hazard ratio and 95% confidence interval for the development of the different endpoints following LAAO procedure. In several steps, we gradually added to the unadjusted model selected demographic characteristics, relevant comorbidities, and risk factors considered to be potential confounders for the primary endpoints All statistical analyses were performed with SPSS/PC statistical software, version 28.0. A *p*-value of less than 0.05 was considered to indicate statistical significance.

## 3. Results

Included in the study were 389 patients with a mean age of 77 ± 7 years who underwent transcatheter LAAO between January 2016 and June 2021, in 18 medical centers throughout Israel. Baseline characteristics of the study population are presented in Table 1. Not surprisingly, participants presented with high rates of previous cardiovascular disease and other non-cardiac comorbidities. The annual performance rate of transcatheter LAAO increased significantly during the study period.

**Figure 1:**
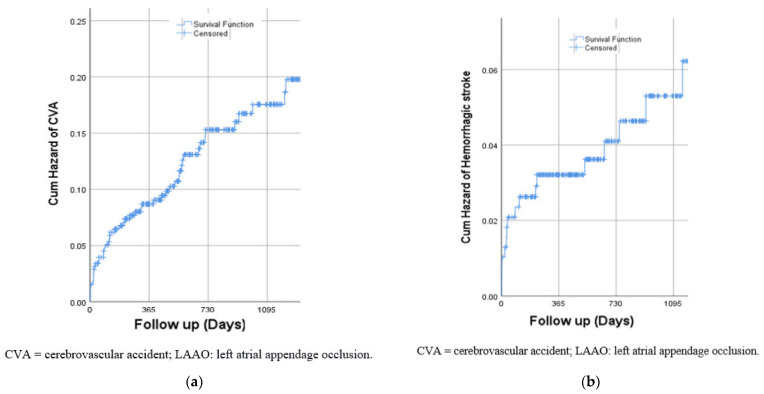
(**a**) Risk of ischemic CVA following LAAO; (**b**) Risk of hemorrhagic CVA following LAAO.

During a median follow-up of 2.1 ± 1.5 years, 52 (13%) patients had ischemic CVA (median time of 7 months, interquartile range: 3.5–19 months) and 17 (4.4%) patients had hemorrhagic CVA (median time of 2.9 months, interquartile range: 0.7–17 months). While the risk of ischemic stroke increased gradually over time (Figure 1a), the risk of hemorrhagic CVA was highest during the first 3 months following the procedure (Figure 1b) with 35% and 53% of cases occurring within the first month and 3 months, respectively. We further conducted a multivariate adjustment for all relevant baseline characteristics. Previous ischemic stroke was the only significant predictor for both hemorrhagic and ischemic stroke (OR = 5.1; 95% CI 2.7–9.6, *p* < 0.001, and OR = 5.8, 95% CI 1.9–17.9, *p* = 0.002, for ischemic and hemorrhagic stroke, respectively).

For the secondary endpoints, 93 (23%) patients died (median time of 17 months, interquartile range: 7.6–30 months), 14 (3%) had myocardial infarction (median time of 29 months, interquartile range: 14–44 months) and 34 (8%) had gastrointestinal bleeding (median time of 4 months, interquartile range: 0.2–14 months). Similarly to hemorrhagic stroke, 50% of GI bleeding events occurred within the first 3 month following the procedure.

## 4. Discussion

The current study is a relatively large report of a multicenter real-life experience with transcatheter LAAO, including patients with non-valvular atrial fibrillation and a contra-indication for anti-coagulation. Not surprisingly, most patients were >75 years old and presented with high rate of previous cardiovascular diseases including stroke. We demonstrated that post procedural long-term prognosis remains poor with a substantial risk of both thrombotic and bleeding events.

The safety and efficacy of transcatheter LAAO have been investigated in several RCTs that demonstrated its non-inferiority compared to anticoagulation [4,5,6]. Accordingly, transcatheter LAAO has been recommended for thromboembolic stroke prevention in patients with atrial fibrillation who cannot be treated with anticoagulation. The PROTECT AF study included 707 AF patients (mean age = 71 ± 9 years, mean CHADS2 score of 2.2 ± 1.2) who were enrolled for LAAO (463 patients) or treatment with warfarin (244 patients). During a mean (SD) follow-up of 3.8 (1.7) years, the composite endpoint including stroke, systemic embolism, and cardiovascular or unexplained death occurred in 8.4% of patients in the device group [5]. Similar low rates of adverse cardiovascular outcomes have been demonstrated in the PREVAIL study (mean age = 74 ± 7 years, mean CHA2DS2-VASc score of 3.8 ± 1.2) that compared LAAO using the Watchman device (269 patients) and chronic warfarin treatment (138 patients) [4]. The PRAGUE 17 trial enrolled high-risk atrial fibrillation patients (mean age = 73 ± 7 years, mean CHA2DS2-VASc score of 4.7 ± 1.5) that were randomized to receive LAAO (*n* = 201) or anticoagulation with DOACs (*n* = 201). LAAO was non-inferior to DOAC in preventing major AF-related cardiovascular, neurological, and bleeding events. Among patients selected for LAAO, the annual rate of the primary outcome (the composite of stroke, systemic embolism, cardiovascular death, major or non-major clinically relevant bleeding, or procedure-related complications) was 11% [6]. In the current study, we demonstrated substantial rates of adverse outcomes, including cardiovascular outcomes, mortality and bleeding in patients undergoing transcatheter LAAO that appear to be higher compared to the randomized controlled trials (Table 2). 

The higher rate of adverse outcomes compared to the randomized controlled trials has several possible explanations. First, our cohort included patients who were older (mean age of 77 ± 7 years) and presented with higher CHA2DS2-VASc score (5 ± 1.5) and higher rate of previous cardiovascular disease including stroke. Indeed, a history of ischemic stroke was the only significant predictor for both hemorrhagic and ischemic stroke. Second, most randomized controlled trials are conducted in selected large-volume hospitals by highly trained teams that have gained substantial experience with the procedure. The current study presents real-life experience from 18 hospitals with different procedural volume and experience. As with all new procedures, there is a learning curve that may result in a higher complications rate in the early period. Accordingly, the association between high hospital procedural volume and better outcomes for LAAO procedures has been well demonstrated [7]. We found a substantial increase in the annual performance rate of transcatheter LAAO in Israel over the last years. Future studies may investigate whether this increase in volume will result in improved outcomes. The differences in patients’ outcomes and procedural characteristics and clinical outcomes between the current study and the relevant RCTs underline the importance of adding real life experience results as complementary data [8]. This can allow better patient selection for each treatment option and improved outcomes. This is even more important since the RCTs on LAAO were tremendously underpowered for individual endpoints of interest and used historically wide non-inferiority margins. 

While the risk of ischemic stroke increased gradually over time, more than a half of hemorrhagic strokes occurred within the first 3 months following the procedure. Current guidelines recommend post-procedural dual anti-platelet therapy with aspirin and clopidogrel for 3 months followed by single anti-platelet therapy (either aspirin or clopidogrel) for life. Whether shortening of dual anti-platelet therapy period or even treatment with single anti-platelet regimens can reduce bleeding risk without increasing thromboembolic risk remains to be evaluated in the future.

The current study has several limitations that warrant consideration. First, the study was conducted in a single country, and therefore, our findings should be extrapolated to other countries with caution. Second, the study did not include a comparison to a control group, since it was impossible to create a relevant group of matched AF patients with a history of significant bleeding, including intracerebral hemorrhage, who have remained on chronic anticoagulation. Third, we did not have data regarding each individual center’s procedural volume and complications rate.

## 5. Conclusions

In conclusion, while the annual performance rate of transcatheter LAAO has increased significantly over the past few years, post-procedural long-term prognosis remains poor with a substantial risk of both thrombotic and bleeding events.

## Figures and Tables

**Table 1:** Baseline characteristics of study participants.

Baseline Characteristics	
*n*	389
Age, years	77 ± 7
Gender, male (%)	248 (63%)
Mean CHA2DS2-VASc Score	5.5 ± 1.5
Body mass index (kg/m^2^)	28 ± 4
Hypertension, *n* (%)	371 (95)
Diabetes mellitus, *n* (%)	60 (15)
CKD, *n* (%)	169 44)
Prior MI, *n* (%)	102 (26)
Heart failure, *n* (%)	190 (49)
Prior CVA/TIA, *n* (%)	176 (45)
PVD, *n* (%)	215 (3)

CKD = Chronic Kidney disease; CVA = Cerebrovascular Accident; TIA = Transient Ischemic Attack; PVD = Peripheral Vascular disease.

**Table 2:** A comparison of baseline characteristics and clinical outcomes of the current study and the relevant randomized controlled trials.

	Prevail Study	Protect AF Study	Prague 17 Study	Our Cohort
Participants	269	463	201	389
Baseline characteristics				
Mean age	74	71	73	77
Mean CHADS2-VASc Score	3.8	2.2 (CHADS Score)	4.7	5.5
Mean follow up	11.8 months	45.6 months	20.8 months	22.8 months
Clinical endpoints				
Ischemic Stroke	1.9%	1.4%	4.4%	13%
Hemorrhagic stroke	0.4%	0.2%	-	4.4%
Major bleeding	0.4%	4.8%	-	8% (GI Bleeding)
All cause death	2.6%	1%	5.4% (CV Death)	23%

GI Bleeding = Gastrointestinal bleeding; CV Death = Cardiovascular death.

## Data Availability

Not applicable.
